# Caregiver Strain and Girls’ ADHD, ODD, and Personality Pathology Symptoms: A One-Year Prospective Study

**DOI:** 10.1007/s10802-026-01472-9

**Published:** 2026-06-13

**Authors:** Helena F. Alacha, Daniel A. Waschbusch, Dara E. Babinski

**Affiliations:** Department of Psychiatry and Behavioral Health, Penn State College of Medicine, Hershey, PA USA

**Keywords:** Caregiver strain, ADHD, ODD, Borderline personality features, Adolescent girls, Longitudinal psychopathology

## Abstract

Caregiver strain influences service utilization and engagement. Prior work—largely centered on boys—shows that attention-deficit/hyperactivity disorder (ADHD) and oppositional defiant disorder (ODD) strongly contribute to strain; however, less is known about these relations in girls. This study examined associations between youth psychopathology symptom dimensions (ADHD, ODD, borderline personality features [BPF], a problem more commonly identified in girls) and caregiver strain facets (objective, subjective internalized, subjective externalized, total strain) among caregivers of 10- to 15-year-old girls (*N* = 197, *M*_*age*_ = 12.06), and whether these associations changed over one year. ADHD, ODD, BPF, and subjective externalized strain increased over time (Δ*M*_*range*_ *=* 0.03-0.36, 95% CIs [0.02, 0.50], *p* ≤ .007, *d* = 0.06-0.15), whereas total and subjective internalized strain decreased (Δ*M*_*range*_ *= −* 0.19 to − 0.05, 95% CIs [-0.21, − 0.03], *p* < .001, *d* = 0.08-0.24). All psychopathology dimensions were positively associated with each caregiver strain facet, concurrently and longitudinally. Linear mixed models revealed positive, facet-specific main effects: ADHD, ODD, and BPF on total and subjective internalized strain; ADHD and BPF on objective strain; and ODD and BPF on subjective externalized strain. A Time × ADHD interaction indicated a weakening association between ADHD and objective strain over time (*b* = − 0.24, 95% CI [-0.43, − 0.05], *p*_FDR_ = 0.022), whereas Time × ODD interactions indicated strengthening associations with total, objective, and subjective externalized strain (*b*_*range*_ = 0.25-0.27, 95% CIs [0.05, 0.48], *p*_FDR_ ≤ 0.032). Findings underscore domain-specific and developmentally dynamic associations between youth psychopathology and caregiver strain in families of adolescent girls.

**Supplementary Information:**

The online version contains supplementary material available at 10.1007/s10802-026-01472-9.

Caregiver strain is a leading reason families seek mental health services for youth with behavioral concerns (Brannan et al. 2003; Bussing et al. 2003a, b b; Gordon and Hinshaw 2017a; Maríñez-Lora et al. 2021; Meltzer et al. 2011; Rice et al. 2020). Although caregiver strain can facilitate help-seeking, higher levels are also associated with greater treatment dropout (Brannan et al., 2003; Burnett-Zeigler & Lyons, 2010; Duppong Hurley et al., 2017), potentially undermining sustained care. Caregiver strain encompasses both observable and emotional burdens associated with caring for a child with mental or behavioral difficulties and is conceptualized as three related but distinct facets—objective, subjective internalized, and subjective externalized strain (Hoenig & Hamilton, 1966). Objective strain refers to tangible challenges (e.g., routine disruption, financial costs), whereas the two subjective facets capture caregivers’ emotional reactions to these demands: subjective internalized strain reflects inward-directed feelings (e.g., embarrassment, hopelessness), whereas subjective externalized strain reflects outward-directed responses toward the child (e.g., anger, blame; Brannan et al., 1997). Although often treated as a unitary construct, accumulating evidence suggests that these facets have differential implications for mental health service outcomes (Accurso et al. 2015; Brannan et al. 2003; Brennan et al. 2022a, b; Frank et al. 2017; Wang and Anderson 2018). For example, objective and subjective internalized strain have been linked to greater service use, higher treatment costs, and more intensive care, whereas subjective externalized strain has been associated with lower service utilization and satisfaction, higher dropout rates, and fewer caregiver employment difficulties (Brannan et al., 2018; Heflinger et al., 2004; Vázquez et al., 2024).

Despite the significance of caregiver strain in treatment seeking and treatment outcome for children, relatively little research has examined caregiver strain among families of girls or how it evolves during potentially vulnerable developmental periods, such as adolescence (Brannan et al., 2003; Frank et al., 2017; Fridman et al., 2017; Green et al., 2020; Tang et al., 2023; Tsai et al., 2015; Vaughan et al., 2013; Wang & Anderson, 2018; Yang et al., 2021). This represents a critical gap, as girls are increasingly identified with attention-deficit/hyperactivity disorder (ADHD), conduct problems, and co-occurring affective and personality pathology symptoms during adolescence, conditions that often impose substantial strain on caregivers (Farris et al., 2011; Keenan et al., 2010; Reuben, 2024).

Prior work, conducted primarily with caregivers of boys, has identified a range of parent-level (e.g., mental health concerns, including ADHD and depression, single-parent status, and female gender) and child-level (e.g., psychopathology severity, number of comorbidities, poor medication adherence, male sex, and age) correlates of caregiver strain (Accurso et al., 2015; Casado-Mejía & Ruiz‐Arias, 2016; Flynn et al., 2023; Tsai et al., 2015; Wang & Anderson, 2018). Across studies, however, youth emotional and behavioral problems consistently emerge as the strongest contributors to strain (Casado‐Mejía & Ruiz‐Arias, 2016; Green et al., 2020; Johnston & Chronis-Tuscano, 2015). Cross-sectional research further indicates that ADHD and co-occurring disruptive behavior disorders, including oppositional defiant disorder (ODD) and conduct disorder (CD), are among the most robust correlates of caregiver strain and are associated with both objective and subjective facets across childhood, adolescence, and young adulthood (Babinski et al. 2020; Brannan et al. 1997; Brennan et al. 2022a, b; Bussing et al. 2003a, b a; Bussing et al. 2003a, b b; Hinojosa et al., 2012; Tang et al., 2023). Although this literature highlights the immediate burden of boys’ externalizing symptoms, it remains unclear whether these patterns persist over time or extend to girls with elevated ADHD and ODD symptoms.

Emerging longitudinal work, also conducted primarily with caregivers of boys, suggests that associations between youth psychopathology and caregiver strain vary over time and are often complex. For instance, in a small sample of adolescent boys with ADHD (*N* = 52, *M*_*age*_ = 11.76), caregiver strain was more strongly associated with absolute levels of conduct problems (composite of ODD and CD) than with changes in these behaviors over one year, although data visualization showed marked heterogeneity across families (Evans et al., 2009). Some families exhibited decreases in both strain and conduct problems over the year, whereas others showed relatively little change in either, suggesting that these patterns may be stable and persistent for some youth. Notably, another subset of parents reported decreases in strain over the year, despite minimal change in conduct problems, potentially reflecting habituation to ongoing behavioral challenges or gradual emotional disengagement from intervention efforts (Evans et al., 2009). These findings highlight the importance of examining how the strength and direction of associations between youth psychopathology and caregiver strain may evolve over time, beyond overall longitudinal trends, particularly among girls, for whom comparable longitudinal data remain limited.

To date, research including girls has been largely cross-sectional and suggests that caregivers of girls report lower levels of strain than caregivers of boys (Brannan and Heflinger 2006; Bussing et al. 2003a, b a; Bussing et al. 2003a, b b). No longitudinal studies have directly examined caregiver strain in girls; however, prospective work following girls with and without ADHD indicates that ADHD symptoms, conduct problems, and affective or interpersonal difficulties contribute to elevated parenting stress (Gordon et al. 2021; Gordon and Hinshaw 2017a, b). Across extended follow-up periods (5-, 10-, and 16-years), overall parenting stress appears to decline among families of girls with and without ADHD (Gordon et al. 2021; Gordon and Hinshaw 2017a, b). Although related, caregiver strain and parenting stress differ in scope and specificity. Parenting stress is a diffuse, global construct that does not clearly distinguish between objective and subjective demands (Abidin, 1992; Deater-Deckard, 1998), whereas caregiver strain more precisely captures distinct emotional and practical burdens in families of youth with behavioral or mental health difficulties (Brannan et al., 1997). Accordingly, longitudinal research is needed to determine whether patterns observed for parenting stress generalize to caregiver strain in girls.

In addition to ADHD and ODD, it is important to examine caregiver strain in relation to other forms of psychopathology that are particularly relevant to girls. Borderline personality features (BPF) frequently co-occur with ADHD and ODD in girls and are thought to reflect shared underlying vulnerabilities, including emotion dysregulation, impulsivity, and interpersonal dysfunction (Babinski et al. 2021a, b; Babinski and McQuade 2019; Brennan et al. 2022a, b; Matthies and Philipsen 2014; Stepp et al. 2012). From a caregiving perspective, BPF may impose qualitatively different and potentially more intensive demands than ADHD or ODD alone. Beyond the behavioral noncompliance typical of externalizing problems, BPF are characterized by affective lability, self-harm, suicidality, interpersonal dysfunction, identity disturbance, and volatile parent-child interactions marked by rapid shifts between dependency and hostility toward caregivers, features that are theoretically and clinically relevant to caregiver strain and family burden (Goodman et al., 2011; Kirtley et al., 2019; Whalen et al., 2014). Although much of the existing literature conceptualizes adolescent BPF as emerging from parenting difficulties, parental psychopathology, or broader family dysfunction (e.g., Armour et al., 2023; Infurna et al., 2016; Kaur & Sanches, 2023; Winsper et al., 2012), it is equally important to consider potential child-to-parent effects of these symptoms. Developmental psychopathology frameworks emphasize that parent-child relations are bidirectional, such that child symptoms may influence caregiver functioning over time (Crnic, 2024; Neville et al., 2025; Olczyk et al., 2024; Pardini, 2008). Emerging evidence indicates that BPF, similar to ADHD and ODD, are associated with both subjective and objective caregiver burden (Bailey & Grenyer, 2013); however, this literature is largely based on adult samples. Consequently, little is known about associations between BPF and caregiver strain in youth.

Adolescence represents a particularly salient developmental period for both the emergence of BPF and the increasing prominence of ADHD and ODD symptoms in a subset of girls (Farris et al., 2011; Sharp & Wall, 2018). Despite evidence that BPF are distressing to families (Jørgensen et al., 2021; Schuppert et al., 2015), few studies have examined their relation to caregiver strain specifically during adolescence or their unique contribution beyond co-occurring psychopathology. Given their affective and interpersonal characteristics, BPF may be particularly likely to elicit subjective facets of caregiver strain, whereas ADHD and ODD may be more strongly associated with objective caregiving demands. Clarifying the associations between caregiver strain and different forms of psychopathology in adolescence is critical for identifying periods of heightened family vulnerability and for guiding the timing and targets of interventions to support girls and their caregivers. In particular, examining how the strength and direction of associations between youth psychopathology symptoms and facets of caregiver strain evolve over time may provide important insight into when families are most vulnerable across development. Additional research in samples of girls with elevated psychopathology symptoms is needed to better understand both concurrent and longitudinal associations between symptom dimensions and caregiver strain in high-risk populations.

## Current Study

The goal of the current study was to examine concurrent and one-year longitudinal associations between youth psychopathology symptom dimensions (ADHD, ODD, and BPF) and facets of caregiver strain in a sample of early adolescent girls overrecruited from outpatient clinical settings for ADHD and related externalizing problems. Based on prior evidence indicating that total caregiver strain and its distinct facets—objective, subjective internalized, and subjective externalized strain—are differentially associated with various domains of youth psychopathology (Frank et al., 2017; Galvan & Gudiño, 2020; Green et al., 2020; Tang et al., 2023; Tsai et al., 2015), total caregiver strain and each strain facet were examined independently.

Aim 1 evaluated mean-level change in youth psychopathology symptoms and caregiver strain over one year; this aim was descriptive, and no directional hypothesis was made given mixed prior evidence regarding whether caregiver strain or youth symptom severity increases, decreases, or remains stable over time (Evans et al. 2009; Gordon and Hinshaw 2017a). Examining these within-variable temporal patterns provides essential developmental context for interpreting between-variable longitudinal associations between youth psychopathology and caregiver strain and helps determine whether these constructs demonstrate stability or change during early adolescence.

Aim 2 examined concurrent and longitudinal associations between youth psychopathology symptoms and caregiver strain. We hypothesized that higher levels of youth psychopathology symptoms would be associated with greater caregiver strain concurrently and longitudinally over one year. Characterizing these between-variable associations establishes the zero-order association structure between youth symptoms and caregiver strain and provides a foundation for subsequent multivariable longitudinal analyses.

Aim 3 assessed the unique associations between youth psychopathology symptoms and caregiver strain while accounting for shared variance among predictors and established child (depression, anxiety, age) and parent (history of depression and ADHD, marital status) correlates (Accurso et al., 2015; Casado-Mejía & Ruiz‐Arias, 2016; Tsai et al., 2015; Wang & Anderson, 2018), and further evaluated whether these associations changed in strength or direction over time. We hypothesized that youth psychopathology symptoms would be uniquely associated with caregiver strain; however, given mixed evidence regarding longitudinal change (Evans et al., 2009), no directional hypotheses were specified for time-dependent effects.

## Method

### Participants

Participants were caregivers of 197 girls who participated in two one-year studies that examined social processing in early adolescent girls. Sample 1 included 87 girls aged 11 to 15 years with (*n* = 44) and without (*n* = 43) ADHD who were originally recruited for a cross-sectional study, although additional funding was obtained and families were contacted one year later to complete additional measures of functioning. Sample 2 included 110 girls aged 10 to 14 years with (*n* = 80) and without (*n* = 30) a history of mental health problems who were recruited for a one-year longitudinal study. The combined sample therefore comprised girls with complex clinical presentations characterized by elevated symptoms of ADHD, ODD, and/or affective and personality pathology. The two samples were combined because they were recruited using similar procedures, drawn from the same outpatient clinical referral context, and included overlapping measures of caregiver strain and youth psychopathology. Importantly, both samples included adolescent girls with substantial variability in externalizing and co-occurring psychopathology, making the integrated sample well-suited to examine concurrent and longitudinal associations between ADHD, ODD, BPF, and facets of caregiver strain. Combining the samples also increased statistical power to detect small-to-moderate effects while maintaining a clinically relevant focus on adolescent girls. Full sociodemographic characteristics are presented in Table 1, and descriptive statistics for psychopathology symptoms and caregiver strain facets are reported in Table 2. Participating families were recruited through local outpatient psychiatric clinics, mailing lists, and social media advertisements. Inclusion criteria required participants to be primary caregivers of girls aged 10–15 years who were residing in the United States and fluent in English. Exclusion criteria included an estimated intelligence quotient below 80 on the Wechsler Abbreviated Scale of Intelligence, Second Edition (Wechsler, 2018), or a history of autism spectrum disorder, bipolar disorder, or a psychotic disorder.

**Table 1:** Sociodemographic characteristics

	M (SD)	*n* (%)
Youth age, years	12.06 (1.24)	
Youth race		
Asian		3 (1.50)
Black and/or African American		5 (2.50)
Multi-racial		13 (6.60)
White		170 (86.30)
Other		3 (1.50)
Missing		3 (1.50)
Youth ethnicity		
Hispanic or Latino		13 (7.10)
Youth lifetime KSADS-derived diagnostic classifications		
ADHD		58 (29.70)
Anxiety disorder		52 (26.70)
CD		12 (6.20)
Mood disorder		29 (14.70)
ODD		63 (32.30)
Suicidal thoughts and/or behaviors		55 (28.20)
Youth receiving psychiatric medication		57 (28.90)
Caregiver relationship to child		
Biological mother		155 (78.70)
Biological father		34 (17.30)
Adoptive father		3 (1.50)
Stepfather		1 (0.50)
Grandmother		4 (2.00)
Parent age, years	42.17 (6.48)	
Parent race		
Asian		3 (1.50)
Black and/or African American		6 (3.10)
Multi-racial		3 (1.50)
White		180 (92.30)
Other		3 (1.50)
Missing		2 (1.00)
Parent ethnicity		
Hispanic or Latino		13 (7.10)
Family income, *n* (%)		
Under $30,000		57 (29.20)
$30,000 to $49,000		34 (17.40)
$50,000 to $79,999		55 (28.20)
$80,000 to $99,999		19 (9.70)
$100,000 and above		30 (15.40)
Missing		2 (1.00)
Parent marital status		
Married		157 (79.70)
Divorced		18 (9.10)
Separated but not divorced		2 (1.00)
Living with a partner		14 (7.10)
Single, never married		5 (2.50)
Widowed		1 (0.50)
Parent education		
High school graduate or equivalency diploma (GED)		14 (17.10)
Some college or trade school		20 (10.20)
Two-year college degree		30 (15.20)
Four-year college degree		64 (32.50)
Master’s degree		45 (22.80)
Doctoral degree		24 (12.20)
Parent has a history of ADHD		53 (26.90)
Parent has a history of depression		97 (49.20)

*N* = 197. Youth lifetime K-SADS-derived diagnostic classifications were assessed via parent report using the computerized Schedule for Affective Disorders and Schizophrenia for School-Aged Children. Anxiety disorder includes panic disorder, social anxiety disorder, generalized anxiety disorder, and/or obsessive-compulsive disorder. Mood disorder includes major depressive disorder, persistent depressive disorder, and/or disruptive mood dysregulation disorder

**Table 2:** Descriptive statistics and mean level change in primary variables from baseline to follow-up

Variable	*Baseline*		*Follow-Up*		t test Comparison			
	M(SD)	Min, Max	M(SD)	Min, Max	ΔM [95% CI]	t(df)	*p*	d
Total caregiver strain	1.63(0.65)	1, 3.75	1.58(0.64)	1, 4	−0.05 [−0.06, − 0.03]	−5.98(6,073)	< 0.001	0.08
Objective caregiver strain	1.56(0.69)	1, 4.20	1.55(0.68)	1, 4.80	−0.01 [−0.03, 0.01]	−1.11(6,073)	0.269	0.01
Subjective internalized caregiver strain	2.01(0.96)	1, 4.75	1.82(0.86)	1, 5	−0.19 [−0.21, − 0.17]	−18.52(6,073)	< 0.001	0.24
Subjective externalized caregiver strain	1.24(0.43)	1, 3	1.33(0.53)	1, 3.67	+ 0.08 [0.07, 0.10]	11.29(6,073)	< 0.001	0.15
ADHD symptoms	0.76(0.69)	0, 2.67	0.79(0.64)	0, 2.65	+ 0.03 [0.02, 0.04]	5.33(6,069)	< 0.001	0.07
ODD symptoms	0.70(0.66)	0, 3	0.75(0.62)	0, 2.88	+ 0.04 [0.03, 0.05]	7.13(6,069)	< 0.001	0.09
BPF	22.82(7.15)	11, 44	22.18(6.64)	11, 42	+ 0.36 [0.22, 0.50]	5.00(6,073)	0.007	0.06
Depression symptoms	7.44(8.10)	0, 45	–	–	–	–	–	–
Anxiety symptoms	14.42(11.47)	0, 49	–	–	–	–	–	–

BPF, depression, and anxiety values reflect means of total scores used in the primary analyses; all other variables reflect mean item scores. Depression and anxiety were assessed at baseline only. Descriptive statistics (*M*, *SD*) and *t* test results are based on imputed data. Unstandardized mean differences (Δ*M*) represent change from baseline to follow-up (*M*₂ − *M*₁). Inferential statistics (*t*, *p*, 95% CI) were pooled across imputations using Rubin’s rules

### Procedure

The Penn State University Institutional Review Board approved all procedures. A phone screen was conducted to establish initial eligibility. Participants had the option to complete consent/assent procedures remotely with a study team member via HIPAA-compliant Microsoft Teams/Zoom prior to the scheduled in-person visit, or during the in-person visit. Written informed consent and assent were obtained electronically from caregivers and participating youth, respectively, via REDCap using e-consent forms. Following caregiver informed consent, youth assent was obtained prior to participation. At baseline, caregivers completed a demographic questionnaire assessing caregiver, child, and family characteristics, as well as the computerized Schedule for Affective Disorders and Schizophrenia for School-Aged Children (KSADS-COMP) to assess lifetime mental disorder symptoms and diagnostic criteria in youth (Townsend et al., 2020). Caregivers and youth also completed measures assessing youth psychopathology and functioning, which were re-administered one year later at the follow-up visit. Families received compensation upon completing the follow-up assessment.

### Measures

#### Youth ADHD and ODD Symptoms

Caregiver report on the Disruptive Behavior Disorders Rating Scale (DBDRS; Pelham et al., 1992) was used to assess youth ADHD symptoms (18 items; baseline: ω = 0.95, α = 0.94; follow-up: ω = 0.95, α = 0.94) and ODD symptoms (8 items; baseline: ω = 0.94, α = 0.92; follow-up: ω = 0.93, α = 0.91). Items were rated on a 4-point scale from 0 (*not at all*) to 3 (*very much*) and averaged, with higher scores reflecting greater symptom severity. Given the high intercorrelations between inattentive and hyperactive/impulsive dimensions across baseline and follow-up (*rs* ≥ 0.76), a total ADHD score was used to reduce concerns regarding multicollinearity and improve the interpretability and stability of parameter estimates (Tabachnick & Fidell, 2019; Cohen, 2013). Although the DBDRS includes a CD subscale (15 items), it was excluded due to zero variance on several items. The ADHD and ODD subscales of the DBDRS have demonstrated excellent internal consistency (α = 0.92–97) across both clinical and community youth samples (Fosco et al., 2023; Silva et al., 2005).

#### Youth BPF

Caregiver report on the Borderline Personality Features Scale for Children—11 (BPFS-P-11; Babinski et al. 2021a, b) was used to assess youth BPF (11 items) at baseline (ω = 0.93, α = 0.86) and follow-up (ω = 0.92, α = 0.85). Items were rated on a 5-point Likert scale from 1 (*not at all true*) to 5 (*always true*) and summed, with higher scores reflecting greater BPF severity. The BPFS-P-11 has demonstrated good to excellent internal consistency in adolescent samples (α = 0.88–93), comparable to that of the original 24-item version (Babinski et al. 2021a, b; Sharp et al. 2011, 2014).

#### Youth Depressive Symptoms

Caregiver report on the Mood and Feelings Questionnaire (MFQ; Costello & Angold, 1988) was used to assess youth depressive symptoms at baseline (33 items; ω = 0.94, α = 0.92). Items were rated on a 3-point scale from 0 (*not true*) to 2 (*true*) and summed, with higher scores reflecting greater depressive symptom severity. The MFQ has demonstrated excellent internal consistency (α = 0.90–94) across both clinical and community youth samples (Costello & Angold, 1988; Jeffreys et al., 2016).

#### Youth Anxiety Symptoms

Caregiver report on the Screen for Child Anxiety Related Emotional Disorders (SCARED; Birmaher et al., 1999) was used to assess youth anxiety symptoms at baseline. Although the SCARED assesses multiple anxiety domains, the present study used the total anxiety score (41 items; ω = 0.94, α = 0.93) as a global index of anxiety severity. Items were rated on a 3-point scale from 0 (*not true or hardly ever true*) to 2 (*very true or often true*) and summed, with higher scores reflecting greater anxiety symptom severity. The SCARED has demonstrated acceptable to excellent internal consistency (α = 0.74–93) in clinical youth samples (Birmaher et al., 1997, 1999).

#### Caregiver Strain

Caregiver report on the Caregiver Strain Questionnaire—Short Form 11 (CGSQ-SF11; Brennan et al. 2022a, b) was used to assess objective strain (5 items; baseline: ω = 0.87, α = 0.83; follow-up: ω = 0.91, α = 0.86), subjective internalized strain (4 items; baseline: ω = 0.91, α = 0.89; follow-up: ω = 0.93, α = 0.92), subjective externalized strain (3 items; baseline: ω = 0.76, α = 0.63; follow-up: ω = 0.81, α = 0.78), and total caregiver strain (12 items; baseline: ω = 0.93, α = 0.91; follow-up: ω = 0.96, α = 0.94). Although the CGSQ-SF11 consists of 11 items, one additional item (“In general, how much of a toll do your child’s problems take on your family?”) was incorporated into the objective and total strain subscales because data were drawn from a measurement-based care initiative (Waschbusch et al., 2020). Items were rated on a 5-point scale from 1 (*not at all*) to 5 (*very much a problem*) and averaged, with higher scores reflecting greater caregiver strain. The CGSQ-SF11 has demonstrated acceptable to excellent internal consistency (α = 0.72–96) in youth samples, comparable to that of the original 21-item CGSQ (Brennan et al. 2022a, b; Göldel and Warschburger 2024).

### Data Analytic Plan

Data were analyzed using SPSS (v31), except where otherwise noted. Attrition was evaluated using two-tailed independent-samples *t* tests and chi-square tests to compare families retained versus lost to follow-up on baseline sociodemographic characteristics (youth and parent age and race/ethnicity; parent relationship to the child; parent marital status; parent education; income; number of siblings), clinical characteristics (youth psychopathology symptoms; youth psychiatric medication status; caregiver strain facets; parent history of ADHD and depression), and sample membership. Differences in overall missingness between Samples 1 and 2 were examined using a chi-square test; incidence rate ratios (IRRs) with 95% confidence intervals (CI) were also computed. Effect sizes were interpreted using Cohen’s *d* for *t* tests (small = 0.20, medium = 0.50, large = 0.80; Cohen, 1992) and phi (φ)/Cramér’s V for chi-square tests (small = 0.10, medium = 0.30, large = 0.50; Cohen, 1988). Outliers were inspected via boxplots. Multicollinearity was assessed using variance inflation factors (VIFs), with values ≥ 5 indicating potential concern (Field, 2018). Missing data were handled using multiple imputation by chained equations (MICE), which reduces bias and increases statistical power to detect small-to-moderate effects (Woods et al., 2024). Imputation models included all analysis variables.

Following imputation, paired-samples *t* tests were conducted to characterize unadjusted mean-level differences in youth psychopathology symptoms (ADHD, ODD, BPF) and caregiver strain facets from baseline to follow-up (Aim 1). Bivariate correlations examined concurrent (baseline, follow-up) and longitudinal associations among primary variables from baseline to follow-up (Aim 2).

Linear mixed models (LMMs) were estimated in R using the *lme4* package (Bates et al., 2015) to examine unique associations between youth psychopathology symptoms (ADHD, ODD, BPF) and caregiver strain facets, and whether these associations changed over time (Aim 3). Models included a random intercept to account for within-person dependence across repeated observations, with time (baseline vs. follow-up) specified as a within-subject factor and youth psychopathology symptoms treated as time-varying predictors. Covariates (youth depression and anxiety symptoms, youth age, parent history of ADHD and depression, parent marital status) were selected a priori to account for established correlates of caregiver strain (Accurso et al., 2015; Casado-Mejía & Ruiz‐Arias, 2016; Tsai et al., 2015; Wang & Anderson, 2018) and to isolate the unique effects of the focal predictors.

Main effects of time tested overall mean-level changes in caregiver strain over time, whereas main effects of youth psychopathology tested associations with caregiver strain averaged across time. Interaction terms between time and youth psychopathology (Time × ADHD, Time × ODD, Time × BPF) tested whether these associations varied in magnitude or direction over time. Significant interactions were probed using simple slopes analyses to estimate (a) overall associations between youth psychopathology and caregiver strain at each time point and (b) changes in caregiver strain over time at low (− 1 SD), mean, and high (+ 1 SD) levels of the predictor. Interactions were visualized using continuous plots of model-estimated associations between youth psychopathology and caregiver strain over time, and simple slopes plots of predicted caregiver strain at baseline and follow-up at each level of the predictor (± 1 SD, mean), adjusted for covariates.

To address multiple comparisons, false discovery rate (FDR) correction using the Benjamini–Hochberg procedure was applied a priori to inferential tests of primary predictors and interaction effects (Aim 3). Consistent with recommendations, FDR correction was not applied to descriptive tests (preliminary analyses, Aim 1) or to secondary parameters supporting subsequent analyses (Aim 2; (Benjamini & Hochberg, 1995; Benjamini & Yekutieli, 2005; Jafari & Ansari-Pour, 2018; Streiner & Norman, 2011; Woods et al., 2024). For transparency, both unadjusted and FDR-corrected *p* values are reported in Table 3; however, only FDR-corrected *p* values (*p*_FDR_) are reported in the text.

**Table 3:** Longitudinal correlations between primary variables at baseline and follow-up

Variable	1.	2.	3.	4.	5.	6.	7.
1. ADHD symptoms	0.83*	0.48*	0.54*	0.44*	0.41*	0.46*	0.23*
2. ODD symptoms	0.58*	0.74*	0.58*	0.46*	0.44*	0.45*	0.31*
3. BPF	0.56*	0.51*	0.67*	0.36*	0.34*	0.38*	0.19*
4. Total caregiver strain	0.61*	0.50*	0.52*	0.54*	0.48*	0.56*	0.36*
5. Objective caregiver strain	0.58*	0.45*	0.45*	0.48*	0.44*	0.48*	0.31*
6. Subjective internalized caregiver strain	0.61*	0.50*	0.54*	0.57*	0.49*	0.60*	0.35*
7. Subjective externalized caregiver strain	0.32*	0.31*	0.28*	0.30*	0.24*	0.29*	0.31*

Values are based on imputed data. Correlations are between baseline variables (left column) and follow-up variables (top row)

*p <.001

## Results

### Preliminary and Attrition Analyses

Missing data analyses indicated that 18.8% of participants who completed baseline (*n* = 37) did not complete the follow-up assessment, consistent with attrition rates observed in longitudinal developmental research (Nicholson et al., 2017). Youth depression symptoms were significantly higher among participants lost to follow-up (*M* = 10.16, *SD* = 10.04, *n* = 37) compared to those retained (*M* = 6.64, *SD* = 7.51, *n* = 160), *t*(195) = 2.40, *p* =.017, Δ*M* = 3.52, 95% CI [0.63, 6.42], *d* = 0.44, reflecting a small effect. No other significant differences in sociodemographic or clinical characteristics were observed (Supplemental Table 1). VIFs (1.76–4.01) for variables included in the primary analyses indicated that multicollinearity was not a concern.

### Mean-Level Changes (Aim 1)

From baseline to follow-up, ADHD symptoms (Δ*M =* 0.03, 95% CI [0.02, 0.04], *p* <.001, *d* = 0.07), ODD symptoms (Δ*M =* 0.04, 95% CI [0.03, 0.05], *p* <.001, *d* = 0.09), BPF (Δ*M =* 0.36, 95% CI [0.22, 0.50], *p* =.007, *d* = 0.06), and subjective externalized strain (Δ*M =* 0.08, 95% CI [0.07, 0.10], *p* <.001, *d* = 0.15) significantly increased, whereas total caregiver strain (Δ*M = −* 0.05, 95% CI [−0.06, − 0.03], *p* <.001, *d* = 0.08) and subjective internalized strain (Δ*M = −* 0.19, 95% CI [−0.21, − 0.17], *p* <.001, *d* = 0.24) significantly decreased; effects were small in magnitude. No other variables significantly changed (Table 2).

### Concurrent and Longitudinal Associations (Aim 2)

Associations between baseline demographics and primary variables varied (Supplemental Table 2). Youth age was negatively associated with ADHD, ODD, BPF, depression, and anxiety symptoms, such that younger youth demonstrated higher levels of these symptoms, but positively associated with total and subjective externalized strain, indicating greater strain among caregivers of older youth. Parent age was positively associated with BPF and depression symptoms, indicating higher levels of these symptoms among youth of older caregivers, and negatively associated with subjective externalized strain, indicating lower levels of strain among older caregivers. Parent histories of depression and ADHD were positively associated with all youth psychopathology symptom dimensions and caregiver strain facets, indicating higher levels among caregivers with these histories. In contrast, parent marital status was negatively associated with all youth psychopathology symptom dimensions and caregiver strain facets, indicating lower levels among married caregivers relative to unmarried caregivers. All youth psychopathology symptom dimensions and caregiver strain facets were positively associated with one another concurrently at baseline (Supplemental Table 2) and follow-up (Supplemental Table 3), as well as longitudinally from baseline to follow-up (Table 4).

**Table 4:** Linear mixed models examining the effects of youth psychopathology on caregiver strain over time

Parameter	b [95% CI]	SE	t	*p*	*p*_FDR_
Dependent variable: Total Caregiver Strain					
Time	− 0.01 [−0.31, 0.29]	0.15	− 0.07	0.945	0.945
History of parent depression	− 0.02 [−0.13, 0.09]	0.06	− 0.36	0.723	0.789
History of parent ADHD	− 0.13 [−0.26, 0.01]	0.07	−1.86	0.063	0.137
Parent marital status	0.03 [−0.11, 0.16]	0.07	0.38	0.707	0.789
Youth age	0.03 [−0.01, 0.07]	0.02	1.52	0.129	0.240
Depression symptoms	0.01 [−0.00, 0.01]	0.00	1.04	0.299	0.486
Anxiety symptoms	0.00 [−0.00, 0.01]	0.00	0.35	0.728	0.789
ADHD symptoms	0.34 [0.22, 0.47]	0.06	5.30	< 0.001	< 0.001
ODD symptoms	0.18 [0.04, 0.32]	0.07	2.49	0.013	0.020
BPF	0.03 [0.01, 0.04]	0.01	3.61	< 0.001	0.001
Time × ADHD symptoms	− 0.16 [−0.32, − 0.00]	0.08	−2.01	0.045	0.054
Time × ODD symptoms	0.27 [0.09, 0.45]	0.09	2.91	0.004	0.008
Time × BPF	− 0.01 [−0.02, 0.01]	0.01	− 0.65	0.515	0.515
*Dependent Variable: Objective Caregiver Strain*					
Time	− 0.05 [−0.40, 0.29]	0.18	− 0.31	0.757	0.981
History of parent depression	− 0.01 [−0.12, 0.10]	0.06	− 0.17	0.866	0.981
History of parent ADHD	− 0.15 [−0.29, − 0.01]	0.07	−2.08	0.039	0.100
Parent marital status	0.02 [−0.12, 0.16]	0.07	0.31	0.758	0.981
Youth age	0.03 [−0.02, 0.07]	0.02	1.22	0.223	0.414
Depression symptoms	0.00 [−0.01, 0.01]	0.01	0.12	0.906	0.981
Anxiety symptoms	0.00 [−0.01, 0.01]	0.00	0.36	0.722	0.981
ADHD symptoms	0.43 [0.29, 0.58]	0.07	6.04	< 0.001	< 0.001
ODD symptoms	0.12 [−0.03, 0.28]	0.08	1.54	0.124	0.149
BPF	0.03 [0.01, 0.04]	0.01	3.21	0.001	0.004
Time × ADHD symptoms	− 0.24 [−0.43, − 0.05]	0.09	−2.55	0.011	0.022
Time × ODD symptoms	0.27 [0.05, 0.48]	0.11	2.44	0.016	0.023
Time × BPF	0.00 [−0.02, 0.02]	0.01	− 0.02	0.856	0.986
*Dependent Variable: Subjective Internalized Caregiver Strain*					
Time	− 0.07 [−0.47, 0.34]	0.21	− 0.33	0.742	0.804
History of parent depression	0.04 [−0.12, 0.19]	0.08	0.47	0.642	0.781
History of parent ADHD	− 0.14 [−0.33, 0.04]	0.10	−1.51	0.133	0.215
Parent marital status	0.02 [−0.17, 0.21]	0.10	0.21	0.837	0.837
Youth age	0.06 [−0.00, 0.11]	0.03	1.92	0.055	0.144
Depression symptoms	0.02 [0.00, 0.03]	0.01	2.61	0.010	0.042
Anxiety symptoms	0.00 [−0.01, 0.01]	0.00	0.44	0.661	0.781
ADHD symptoms	0.40 [0.22, 0.58]	0.09	4.34	< 0.001	< 0.001
ODD symptoms	0.24 [0.05, 0.44]	0.10	2.44	0.015	0.031
BPF	0.03 [0.01, 0.05]	0.01	3.20	0.001	0.004
Time × ADHD symptoms	− 0.18 [−0.40, 0.03]	0.11	−1.67	0.095	0.123
Time × ODD symptoms	0.20 [−0.04, 0.44]	0.12	1.64	0.102	0.123
Time × BPF	− 0.01 [−0.03, 0.02]	0.01	− 0.49	0.623	0.623
*Dependent Variable: Subjective Externalized Caregiver Strain*					
Time	0.06 [−0.23, 0.36]	0.15	0.42	0.673	0.697
History of parent depression	− 0.09 [−0.20, 0.02]	0.06	−1.59	0.114	0.295
History of parent ADHD	− 0.08 [−0.21, 0.05]	0.07	−1.16	0.247	0.491
Parent marital status	0.04 [−0.10, 0.17]	0.07	0.51	0.608	0.697
Youth age	0.01 [−0.03, 0.05]	0.02	0.39	0.697	0.697
Depression symptoms	0.00 [−0.01, 0.01]	0.00	− 0.42	0.674	0.697
Anxiety symptoms	0.00 [−0.01, 0.00]	0.00	−1.12	0.264	0.491
ADHD symptoms	0.10 [−0.02, 0.23]	0.06	1.61	0.108	0.163
ODD symptoms	0.21 [0.07, 0.35]	0.07	2.97	0.003	0.019
BPF	0.02 [0.00, 0.03]	0.01	2.27	0.024	0.048
Time × ADHD symptoms	− 0.07 [−0.24, 0.09]	0.08	− 0.88	0.380	0.455
Time × ODD symptoms	0.25 [0.06, 0.43]	0.10	2.57	0.011	0.032
Time × BPF	− 0.01 [−0.02, 0.01]	0.01	− 0.74	0.458	0.458

Values are based on imputed data

### Change in Associations Over Time (Aim 3)

#### Total Caregiver Strain

**Fig. 1:**
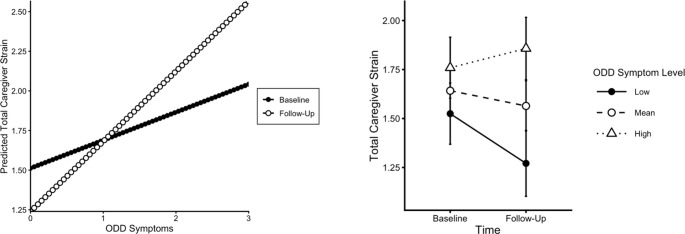
Time × ODD symptoms interaction predicting total caregiver strain. *Note. *The left panel illustrates the Time × ODD symptoms interaction, showing predicted levels of total caregiver strain across the full range of youth ODD symptoms at baseline and follow-up. The right panel displays estimated marginal means of total caregiver strain as a function of time (baseline vs. follow-up) at low (−1 SD), mean, and high (+1 SD) levels of ODD symptoms. Error bars represent ±1 SE

Significant main effects of youth ADHD symptoms and BPF emerged, such that higher levels of ADHD symptoms and BPF were associated with greater total caregiver strain (Table 3). A significant main effect of youth ODD symptoms also emerged; however, this effect was qualified by a significant Time × ODD interaction, indicating that the association between ODD symptoms and total caregiver strain strengthened over time (Fig. 1). Simple slopes analyses indicated that the association between ODD symptoms and total caregiver strain was significant at both time points and increased in magnitude from baseline (*b* = 0.18, 95% CI [0.03, 0.32], SE = 0.07, *t* = 2.44, *p*_FDR_ = 0.015) to follow-up (*b* = 0.44, 95% CI [0.29, 0.60], SE = 0.08, *t* = 5.65, *p*_FDR_ < 0.001). Probing this interaction at low (− 1 SD), mean, and high (+ 1 SD) levels of ODD symptoms showed that total caregiver strain decreased significantly over time at low (*b* = − 0.26, 95% CI [−0.40, − 0.11], SE = 0.07, *t* = −3.49, *p*_FDR_ < 0.001) and mean (*b* = − 0.08, 95% CI [−0.16, − 0.01], SE = 0.04, *t* = −2.14, *p*_FDR_ = 0.032) levels of ODD symptoms, but did not change significantly at high levels of ODD symptoms (*b* = 0.09, 95% CI [−0.04, 0.23], SE = 0.07, *t* = 1.34, *p*_FDR_ = 0.180; Fig. 1). A Time × ADHD interaction was also observed (*p* =.045); however, this effect did not remain significant after FDR correction (*p*_FDR_ = 0.054). No significant main effect of time was observed.

#### Objective Caregiver Strain

**Fig. 2:**
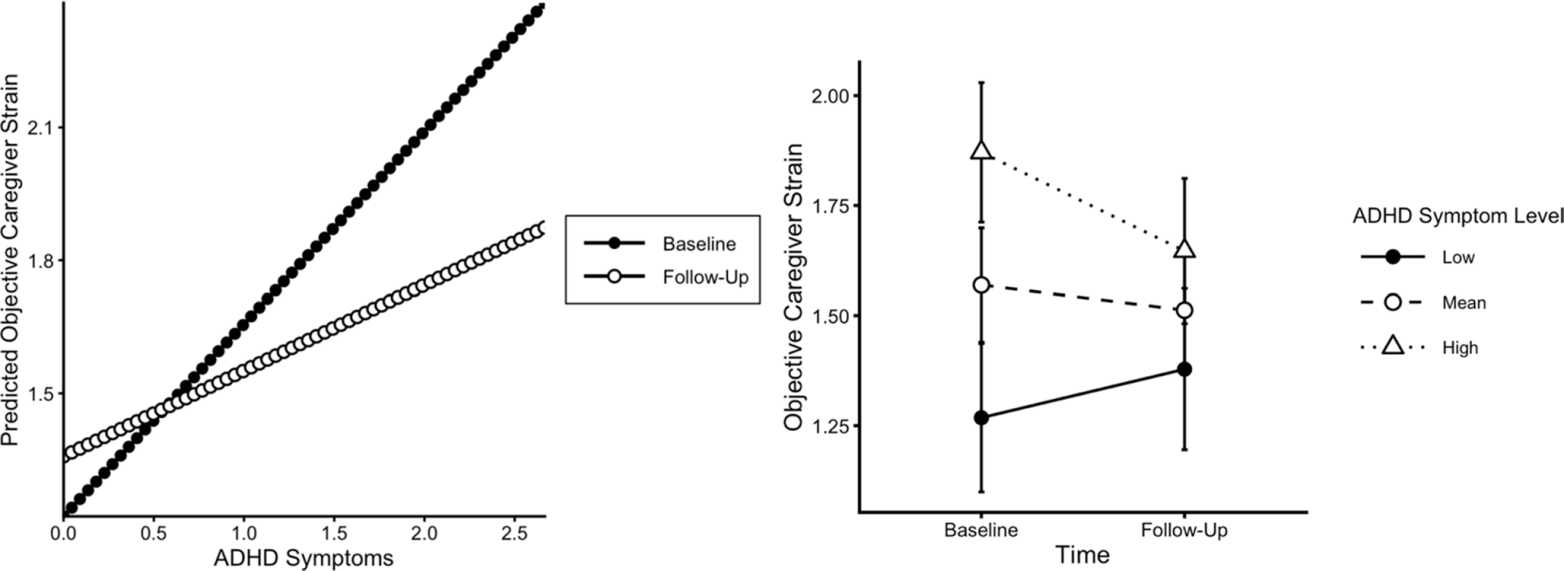
Time × ADHD symptoms interaction predicting objective caregiver strain. *Note.* The left panel illustrates the Time × ADHD symptoms interaction, showing predicted levels of objective caregiver strain across the full range of youth ADHD symptoms at baseline and follow-up. The right panel displays estimated marginal means of objective caregiver strain as a function of time (baseline vs. follow-up) at low (−1 SD), mean, and high (+1 SD) levels of ADHD symptoms. Error bars represent ±1 SE

Significant main effects of youth ADHD symptoms and BPF emerged, such that higher levels of ADHD symptoms and BPF were associated with greater objective caregiver strain (Table 3). A significant Time × ADHD interaction emerged, indicating that the association between ADHD symptoms and objective strain weakened over time (Fig. 2). Simple slopes analyses indicated that the association between ADHD symptoms and objective caregiver strain was positive at both time points but decreased in magnitude from baseline (*b* = 0.43, 95% CI [0.29, 0.58], SE = 0.07, *t* = 5.92, *p*_FDR_ < 0.001) to follow-up (*b* = 0.19, 95% CI [0.03, 0.35], SE = 0.08, *t* = 2.39, *p*_FDR_ = 0.017). Probing this interaction at low (− 1 SD), mean, and high (+ 1 SD) levels of ADHD symptoms showed that objective strain did not change significantly over time at low (*b* = 0.11, 95% CI [−0.05, 0.27], SE = 0.08, *t* = 1.39, *p*_FDR_ = 0.165) or mean (*b* = − 0.05, 95% CI [−0.14, 0.03], SE = 0.04, *t* = −1.22, *p*_FDR_ = 0.221) levels of ADHD symptoms, but decreased significantly over time at high levels of ADHD symptoms (*b* = − 0.22, 95% CI [−0.37, − 0.07], SE = 0.08, *t* = −2.85, *p*_FDR_ = 0.004; Fig. 2). A significant Time × ODD interaction was also observed, indicating that the association between youth ODD symptoms and objective strain strengthened over time (Supplemental Fig. 1), mirroring the pattern observed for total caregiver strain. Simple slopes analyses indicated that the association between ODD symptoms and objective strain was not significant at baseline (*b* = 0.12, 95% CI [−0.03, 0.28], SE = 0.08, *t* = 1.52, *p*_FDR_ = 0.127), but was significant at follow-up (*b* = 0.36, 95% CI [0.20, 0.52], SE = 0.08, *t* = 4.33, *p*_FDR_ < 0.001). Probing this interaction at low (− 1 SD), mean, and high (+ 1 SD) levels of ODD symptoms showed that objective caregiver strain decreased significantly over time at low levels of ODD symptoms (*b* = − 0.24, 95% CI [−0.41, − 0.07], SE = 0.09, *t* = −2.74, *p*_FDR_ = 0.006), but did not change significantly at mean (*b* = − 0.06, 95% CI [−0.15, 0.02], SE = 0.04, *t* = −1.40, *p*_FDR_ = 0.163) or high (*b* = 0.11, 95% CI [−0.05, 0.28], SE = 0.08, *t* = 1.36, *p*_FDR_ = 0.175) levels of ODD symptoms (Supplemental Fig. 1). No significant main effect of time was observed.

#### Subjective Internalized Caregiver Strain

Significant main effects of youth ADHD symptoms, ODD symptoms, BPF, and depression symptoms emerged, such that higher levels of these symptoms were associated with greater subjective internalized caregiver strain (Table 3). No significant main effect of time or interactions between time and youth symptom dimensions were observed.

#### Subjective Externalized Caregiver Strain

A significant main effect of youth BPF emerged, such that higher levels of BPF were associated with greater subjective externalized caregiver strain (Table 3). A significant main effect of youth ODD symptoms also emerged; however, this effect was qualified by a significant Time × ODD interaction, indicating that the association between ODD symptoms and subjective externalized strain strengthened over time (Supplemental Fig. 2), mirroring the pattern observed for total caregiver strain. Simple slopes analyses indicated that the association between ODD symptoms and subjective externalized strain was significant at both time points and increased in magnitude from baseline (*b* = 0.21, 95% CI [0.07, 0.36], SE = 0.07, *t* = 2.92, *p*_FDR_ = 0.004) to follow-up (*b* = 0.46, 95% CI [0.29, 0.62], SE = 0.08, *t* = 5.47, *p*_FDR_ < 0.001). Probing this interaction at low (− 1 SD), mean, and high (+ 1 SD) levels of ODD symptoms showed that subjective externalized caregiver strain did not change significantly over time at low (*b* = − 0.13, 95% CI [−0.28, 0.02], SE = 0.08, *t* = −1.70, *p*_FDR_ = 0.089) or mean (*b* = 0.03, 95% CI [−0.04, 0.11], SE = 0.04, *t* = 0.89, *p*_FDR_ = 0.375) levels of ODD symptoms, but increased significantly over time at high levels of ODD symptoms (*b* = 0.20, 95% CI [0.05, 0.34], SE = 0.07, *t* = 2.71, *p*_FDR_ = 0.007; Supplemental Fig. 2). No significant main effect of time was observed.

## Discussion

This study is among the first to examine how youth psychopathology symptom dimensions (ADHD, ODD, BPF) uniquely and dynamically relate to facets of caregiver strain in families of early adolescent girls. All youth psychopathology symptom dimensions were positively associated with caregiver strain facets in bivariate analyses, both concurrently and longitudinally from baseline to follow-up. Multivariable models, however, indicated that these associations varied meaningfully across strain facets when symptom dimensions and covariates were considered simultaneously. ADHD, ODD, and BPF demonstrated main effects on total and subjective internalized strain; ADHD and BPF on objective strain, and ODD and BPF on subjective externalized strain. Notably, the association between ADHD symptoms and objective strain weakened over time, whereas the associations between ODD symptoms and total strain, objective strain, and subjective externalized strain strengthened over time. These findings are discussed herein.

Examination of mean-level change revealed distinct patterns of stability and change across youth psychopathology symptoms and caregiver strain facets over the one-year follow-up. Although prior research suggests heterogeneous developmental trajectories, with some studies documenting declines in symptom severity and others noting increases or persistence during adolescence, particularly for externalizing behaviors and BPF (Biederman, 2000; Larsson et al., 2011; Vergunst et al., 2019; Wright et al., 2016), the present findings indicated significant increases in ADHD, ODD, and BPF across the study period. Caregiver strain demonstrated facet-specific change, such that total and subjective internalized strain decreased, subjective externalized strain increased, and objective strain remained stable. This pattern partially aligns with prior work documenting small-to-moderate declines across all caregiver strain facets over a shorter, eight-month interval in a predominantly male sample (Accurso et al., 2015), but also suggests potentially divergent developmental trajectories in girls regarding subjective externalized and objective strain.

These findings point to a qualitative shift in caregivers’ emotional experience, from inwardly directed distress to more outwardly expressed responses as girls progress through adolescence. This shift may reflect key distinctions between the two subjective strain facets. Subjective internalized strain captures internally experienced emotions (e.g., worry, guilt, sadness), which can naturally fluctuate over time as part of broader adaptive processes (Ashman et al., 2008; Wu, 2024). In contrast, subjective externalized strain reflects more overt expressions of frustration, irritation, or interpersonal conflict, which may intensify during adolescence as youth autonomy increases and parent–child dynamics become more complex (Kiesner et al., 2009; Xie et al., 2021).

This pattern is also consistent with typical developmental changes during adolescence, including increased independence seeking and emotional volatility, particularly among girls (Silverberg & Steinberg, 1987; Zukauskiene, 2017). Although normative, these changes may exacerbate interpersonal strain within the family, especially when oppositional behaviors challenge parental authority or family cohesion (Bailen et al., 2019; Garcia et al., 2019). Increases in subjective externalized strain may reflect heightened interpersonal conflict during this period. In contrast, reductions in subjective internalized strain and total strain may reflect caregiver adaptation or shifts in the appraisal of chronic stressors (even when objective caregiving demands remain similar), consistent with transactional models of caregiver stress and coping (Deater-Deckard, 1998; Pearlin et al., 1981). Accordingly, caregivers may experience less internal distress over time not because demands decreased, but because they adapted or came to view ongoing challenges as more manageable, with changes that were small yet consistent across participants. Importantly, the magnitude of mean-level change was small, suggesting subtle but systematic shifts rather than large changes in caregiver functioning.

As expected, all youth psychopathology symptom dimensions were positively associated with each caregiver strain facet in cross-sectional and longitudinal correlational analyses. LMM results further delineated the unique effects of psychopathology dimensions on specific caregiver strain facets. ADHD, ODD, and BPF were each uniquely associated with total and subjective internalized strain, reflecting broad contributions to overall caregiver burden and heightened internal distress. ADHD and BPF were additionally associated with objective strain, suggesting greater practical caregiving demands, whereas ODD and BPF were associated with subjective externalized strain, indicating greater relational conflict and outward expressions of distress.

Consideration of longitudinal associations between youth psychopathology and caregiver strain provided additional nuance. A significant Time × ODD interaction indicated that the association between ODD symptoms and total strain strengthened over the year. This pattern suggests that oppositional behaviors (e.g., defiance, irritability, conflict with authority) may become increasingly salient contributors to family stress, heightening interpersonal tension and potentially undermining the effectiveness of caregiver strategies (Liu et al., 2018). These effects may be particularly pronounced for girls, as such behaviors often conflict with societal expectations emphasizing obedience, warmth, and empathy (Brody & Hall, 2010). In this context, caregivers may be more likely to interpret oppositional behaviors as reflecting personal parenting failures or moral shortcomings, thereby amplifying both frustration and self-directed distress. Additionally, the slightly higher number of youth with ODD-consistent clinical profiles (*n* = 63) compared to ADHD (*n* = 58) in the present sample, as assessed via the K-SADS-COMP, may further underscore the prominence of ODD-related strain, consistent with evidence that ODD symptoms are especially burdensome for families (Meltzer et al., 2011).

Analyses of caregiver strain facets revealed additional specificity. A significant Time × ADHD interaction indicated that the association between ADHD symptoms and objective strain weakened over time. This pattern suggests that caregivers may develop routines or coping strategies that reduce the practical demands of managing ADHD, even as symptoms persist or worsen. In contrast, significant Time × ODD interactions indicated that associations between ODD and both objective and subjective externalized strain strengthened over time. This pattern is broadly consistent with coercive family process models (Patterson et al., 1989), which posit reciprocal reinforcement between child behavior and caregiver responses that may amplify stress over time. Longitudinal findings from the Pittsburgh Girls Study similarly demonstrate these processes in girls, with oppositional and antagonistic behavior predicting increases in caregiver verbal aggression, which in turn predicts subsequent elevations in youth irritability and antagonism (Derella et al., 2020). Together, these findings suggest that ODD-related strain may escalate over time as maladaptive interaction patterns become more entrenched, whereas ADHD-related strain may stabilize or attenuate as caregivers adapt, highlighting the importance of targeting distinct mechanisms across disorders.

The present findings extend a literature that has largely focused on boys. Consistent with previous findings, both externalizing and internalizing symptoms were associated with caregiver strain, with ODD symptoms emerging as the most robust (Evans et al., 2009; Frank et al., 2017; Tsai et al., 2015; Vaughan et al., 2013). These parallels suggest that core associations between youth behavioral and emotional dysregulation and caregiver distress may generalize across sex. At the same time, several patterns warrant consideration. Prior research has predominantly examined overall caregiver strain or parental stress, rather than its distinct facets, and has yielded mixed evidence regarding its trajectory (Borre & Kliewer, 2014; Dijk et al., 2022; Evans et al., 2009; Gordon et al., 2021). Studies of total caregiver strain often demonstrate substantial individual-level variability, with opposing changes offsetting one another and resulting in minimal group-level effects (Evans et al., 2009; Lindly et al., 2022; Townsend et al., 1989).

The limited facet-specific work has also produced inconsistent results. For instance, caregivers of children with disruptive behavior problems (*M*_*age*_ = 9.0, 67.9% male) reported reductions across all strain facets over eight months (Accurso et al., 2015), whereas caregivers of children with autism (*M*_*age*_ = 9.7, 80.7% male) did not indicate any meaningful change across facets over a 2- to 5-year period (Lindly et al., 2022). In contrast, the present findings indicate a more nuanced pattern of change among caregivers of adolescent girls, characterized by increases in subjective externalized strain alongside decreases in subjective internalized and total strain, with objective strain remaining stable. Although methodological differences may partially account for inconsistencies, these results suggest that caregiver strain may follow domain-specific trajectories rather than a uniform pattern, particularly in families of girls. Additionally, several established parent (e.g., mental health history, marital status) and child (e.g., anxiety/depression symptoms, age) correlates of caregiver strain identified in predominantly male samples (Accurso et al., 2015; Casado-Mejía & Ruiz‐Arias, 2016; Tsai et al., 2015; Wang & Anderson, 2018) did not emerge in the present study. These findings suggest that associations with caregiver strain may vary by sex and developmental context.

### Limitations and Future Research Directions

Several limitations should be noted. First, data were only collected at two time points across a relatively short follow-up interval, which may have limited the ability to capture nonlinear developmental change and to disentangle within-person from between-person processes over time. More frequent and intensive longitudinal assessments (e.g., daily diary) may better capture short-term fluctuations and transactional associations between youth psychopathology and caregiver strain (Alacha et al., 2026). In addition, although multiple imputation was used to address missing data, the reduced sample size at follow-up may have limited statistical power and precision, particularly for detecting small effects; accordingly, null or small associations should be interpreted cautiously and warrant replication in larger longitudinal samples. Second, the study relied exclusively on caregiver-reported measures of youth psychopathology, which may introduce shared method variance and reporting biases (Alacha & Lefler, 2021; Van Roy et al., 2010). Future research should incorporate multi-informant assessments, including youth reports and independent measures of family functioning, to evaluate cross-reporter consistency and bidirectional processes.

Third, youth depression and anxiety were only assessed at baseline, preventing evaluation of how changes in these symptoms relate to caregiver strain over time. Relatedly, sociodemographic and parent psychopathology were also limited to baseline assessment, despite the potential for fluctuations in these domains over time (Curran & Bauer, 2011). Fourth, although youth were oversampled for mental health concerns, caregiver strain scores were lower than those typically reported in clinical samples (Accurso et al., 2015; Babinski et al., 2020; Brannan et al., 2018; Tsai et al., 2015), and the sample was predominantly White and composed mostly of mothers, limiting generalizability to more diverse or clinically referred populations. Finally, higher baseline depression among participants lost to follow-up may further limit generalizability to girls with greater depressive severity. Future research using repeated assessments in clinically and sociodemographically diverse samples is needed to better delineate trajectories of caregiver strain and identify key risk and protective factors.

### Clinical Implications

The present findings have important clinical implications. Differentiating among caregiver strain facets indicates that distinct types of strain may require targeted, mechanism-specific intervention strategies related to youth psychopathology dimensions. Objective strain, uniquely associated with ADHD and BPF, may be most effectively addressed through structured behavioral management and organizational supports (Tsai et al., 2015). Subjective externalized strain, associated with ODD and BPF, may require interventions targeting family processes and conflict management, such as parent–child interaction therapy or collaborative problem solving. Subjective internalized strain, associated with ADHD, ODD, and BPF, may benefit from interventions targeting cognitive appraisal, stress management, and emotion regulation. Across strain facets, findings underscore the value of integrating caregiver-focused strategies with youth-focused care to reduce strain (Zeiler et al., 2025). Addressing caregiver cognitions, stress responses, and emotion regulation alongside youth symptoms is likely critical for reducing strain and improving overall family functioning (Kazdin & Whitley, 2003; Marçal, 2021; Mendenhall & Mount, 2011). These findings further suggest that intervention needs may shift across development, with adolescence representing a period of increasing interpersonal strain, particularly in the context of oppositional behaviors (Alacha et al., 2024; Stepp et al., 2012). This highlights the importance of maintaining flexibility in intervention approaches to address evolving needs as youth and caregivers progress through different life stages.

For families of girls, incorporating gender-sensitive psychoeducation into treatment may be particularly beneficial. Caregivers may interpret oppositionality or emotional intensity through the lens of gendered expectations, potentially amplifying strain; framing some of these behaviors as developmentally normative may help reduce negative attributions and caregiver distress (Endendijk et al., 2016; Moon & Hoffman, 2008). Importantly, the strengthening associations between ODD symptoms and total, objective, and subjective externalized caregiver strain over time highlight the need for sustained intervention and ongoing support. Periodic booster sessions and continued monitoring may help prevent escalation of oppositional behaviors and cumulative caregiver burden.

More broadly, caregiver strain may function as both a treatment target and a clinically informative marker. Elevated strain often prompts help-seeking, but is also associated with dropout and disengagement (Brannan et al., 2003; Burnett-Zeigler & Lyons, 2010; Duppong Hurley et al., 2017). Routine, facet-specific and gender-sensitive assessment of strain may help identify families at risk for poor engagement and guide treatment adaptation. Multisystemic supports, including school collaboration, community resources, and peer programs, may further reduce objective burdens while helping normalize caregivers’ emotional experiences.

Overall, these findings position caregiver strain as a clinically relevant target for intervention in families of girls with elevated symptoms of ADHD, ODD, and BPF. Developmentally informed, gender-sensitive approaches that simultaneously address youth and caregiver processes are likely to yield the most lasting benefits across adolescence.

## Supplementary Information

Below is the link to the electronic supplementary material.

Supplementary Material 1 (DOCX 2.99 MB)

## Data Availability

Data are available upon reasonable request to the senior author, DEB.
