# Supplementary material for: Caregiver Strain and Girls’ ADHD, ODD, and Personality Pathology Symptoms: A One-Year Prospective Study
Source: Res Child Adolesc Psychopathol. 2026 Jun 13;54(3):76. doi: 10.1007/s10802-026-01472-9 (PMC13264552; doi:10.1007/s10802-026-01472-9)
Supplement: Supplementary file 1 — Supplementary Material 1 (DOCX 2.99 MB) [file 10802_2026_1472_MOESM1_ESM.docx]

**Supplemental Table 1**

*Attrition Analyses for Participants Lost to Follow-up*

| Baseline Characteristic | Follow-Up | | Statistical Comparison | | | |
| --- | --- | --- | --- | --- | --- | --- |
|  | Did Not Complete | Completed |  |  |  |  |
| **Continuous Variables, *M* (*SD*)** | ***N* = 37** | ***N* = 160** | **Δ*M* [95% CI]** | ***t*(df)** | ***p*** | ***d*** |
| ADHD symptoms | .76 (.63) | .76 (.71) | .00 [-.25, .25] | .00(195) | .997 | .00 |
| ODD symptoms | .79 (.69) | .68 (.66) | .11 [-.13, .35] | .94(195) | .351 | .17 |
| CD symptoms | .08 (.13) | .09 (.16) | .01[-.07, .05] | -.19(195) | .852 | .03 |
| BPF | 23.70 (7.95) | 21.39 (6.94) | 2.31 [-.26, 4.89] | 1.77(195) | .078 | .32 |
| Depression symptoms | 10.16 (10.04) | 6.64 (7.51) | 3.52 [.63, 6.42] | 2.40(195) | .017 | .44 |
| Anxiety symptoms | 14.62 (10.78) | 14.39 (11.63) | .23 [-3.90, 4.36] | .11(195) | .911 | .02 |
| Total caregiver strain | 1.70 (.74) | 1.62 (.63) | .08 [-.16, .31] | .67(195) | .507 | .12 |
| Objective caregiver strain | 1.62 (.87) | 1.54 (.65) | .07 [-.18, .32] | .54(195) | .590 | .10 |
| Subjective internalized caregiver strain | 2.05 (.97) | 2.00 (.97) | .04 [-.31, .39] | .23(195) | .819 | .04 |
| Subjective externalized caregiver strain | 1.36 (.46) | 1.23 (.42) | .14 [-.01, .30] | 1.84(195) | .067 | .34 |
| Youth age | 12.27 (1.19) | 12.01 (1.25) | .26 [-.19, .70] | 1.14(195) | .258 | .21 |
| Number of siblings | 1.89 (1.17) | 2.00 (1.51) | .11 [-.63, .42] | -.41(195) | .684 | .07 |
| Parent age | 42.57 (7.29) | 42.08 (6.30) | .49 [-1.84, 2.83] | .42(195) | .493 | .08 |
| **Categorical Variables, *n* (%)** |  |  | **Chi-Square Test** | | | |
| Youth psychiatric medication |  |  |  | | | |
| Not prescribed | 27 (19.30%) | 113 (80.70%) | χ²(1, *N* = 197) = .08, *p* = .777, φ = .02 | | | |
| Prescribed | 10 (17.50%) | 47 (82.50%) |  |  |  |  |
| Youth race |  |  |  | | | |
| Asian | 2 (66.70%) | 1 (33.30%) | χ²(4, *N* = 197) = 4.99, *p* = .288, V = .16 | | | |
| Black and/or African American | 1 (20.00%) | 4 (80.00%) |  |  |  |  |
| White | 31 (18.20%) | 139 (81.80%) |  |  |  |  |
| Multi-ethnic | 2 (15.40%) | 11 (84.60%) |  |  |  |  |
| Other | 1 (33.30%) | 2 (66.70%) |  |  |  |  |
| Youth ethnicity |  |  |  | | | |
| Not Hispanic or Latino | 30 (17.50%) | 141 (82.50%) | χ²(1, *N* = 184) = 3.43, *p* = .064, φ = .14 | | | |
| Hispanic or Latino | 5 (38.50%) | 8 (61.50%) |  |  |  |  |
| Parent race |  |  |  | | | |
| Asian | 2 (66.70%) | 1 (33.30%) | χ²(4, *N* = 195) = 6.41, *p* = .171, V = .18 | | | |
| Black and/or African American | 2 (33.30%) | 4 (66.70%) |  |  |  |  |
| White | 31 (17.20%) | 149 (82.80%) |  |  |  |  |
| Multi-ethnic | 1 (33.30%) | 2 (66.70%) |  |  |  |  |
| Other | 1 (33.30%) | 2 (66.70%) |  |  |  |  |
| Parent ethnicity |  |  |  | | | |
| Not Hispanic or Latino | 31 (17.40%) | 147 (82.60%) | χ²(1, *N* = 188) = 3.19, *p* = .074, φ = .13 | | | |
| Hispanic or Latino | 4 (40.00%) | 6 (60.00%) |  |  |  |  |
| Caregiver/parent relationship |  |  |  | | | |
| Biological mother | 28 (18.10%) | 127 (81.90%) | χ²(4, *N* = 197) = 7.66 *p* = .105, V = .20 | | | |
| Biological father | 6 (17.60%) | 28 (82.40%) |  |  |  |  |
| Adoptive father | 0 (0.00%) | 3 (100%) |  |  |  |  |
| Stepfather | 1 (100%) | 0 (0.00%) |  |  |  |  |
| Grandmother | 2 (50.00%) | 2 (50.00%) |  |  |  |  |
| Parent marital status |  |  |  | | | |
| Not married | 10 (25.00%) | 30 (75.00%) | χ²(1, *N* = 197) = 1.27, *p* = .259, φ = .08 | | | |
| Married | 27 (17.20%) | 130 (82.80%) |  |  |  |  |
| Parent history of ADHD |  |  |  | | | |
| No history | 25 (17.40%) | 119 (82.60%) | χ²(1, *N* = 197) = .71, *p* = .400, φ = .06 | | | |
| Yes history | 12 (22.60%) | 41 (77.40%) |  |  |  |  |
| Parent history of depression |  |  |  | | | |
| No history | 20 (20.00%) | 80 (80.00%) | χ²(1, *N* = 197) = .20, *p* = .657, φ = .03 | | | |
| Yes history | 17 (17.50%) | 80 (82.50%) |  |  |  |  |
| Parent education |  |  |  | | | |
| High school graduate or equivalency | 4 (28.60%) | 10 (71.40%) | χ²(5, *N* = 197) = 3.05, *p* = .693, V = .12 | | | |
| Some college or trade school | 5 (25.00%) | 15 (75.00%) |  |  |  |  |
| Two-year college degree | 5 (16.70%) | 25 (83.30%) |  |  |  |  |
| Four-year college degree | 9 (14.10%) | 55 (85.90%) |  |  |  |  |
| Master’s degree | 8 (17.80%) | 37 (82.20%) |  |  |  |  |
| Doctoral degree | 6 (25.00%) | 18 (75.00%) |  |  |  |  |
| Family income |  |  |  | | | |
| Under $30,000 | 10 (17.50%) | 47 (82.50%) | χ²(4, *N* = 195) = 1.94, *p* = .747, V = .10 | | | |
| $30,000 to $49,000 | 5 (14.70%) | 29 (85.30%) |  |  |  |  |
| $50,000 to $79,999 | 9 (16.40%) | 46 (83.60%) |  |  |  |  |
| $80,000 to $99,999 | 4 (21.10%) | 15 (78.90%) |  |  |  |  |
| $100,000 and above | 8 (26.70%) | 22 (73.30%) |  |  |  |  |
| Sample membership |  |  |  | | | |
| Sample 1 | 21 (24.40%) | 65 (75.60%) | χ²(1, *N* = 197) = 3.18, *p* = .075, φ = .13 | | | |
| Sample 2 | 16 (14.40%) | 95 (85.60%) |  |  |  |  |
| **Missing Data Comparison** | | | | | | |
| Sample 1 missing data % | 24.20% | | IRR = 1.77, 95% CI [.87, 3.69], *p* = .092  χ²(1) = 3.22, *p* = .856, φ = .01 | | | |
| Sample 2 missing data % | 13.60% | |  |  |  |  |

*Note.* Values reflect observed (pre-imputation) data. Two-tailed independent samples *t*-tests compared continuous variables (youth psychopathology symptoms [ADHD, ODD, CD, BPF, depression, anxiety], caregiver strain levels, youth and parent age, number of siblings), and chi-square tests compared categorical variables (youth psychiatric medication status, youth and parent race, youth and parent ethnicity, caregiver relationship to the child, parent marital status, parent history of ADHD and depression, parent education, family income, and sample membership) between participants who completed the follow-up visit and those who were lost to follow-up. In addition, overall missing data rates were compared between Sampe 1 and Sample 2. Reported 95% confidence intervals (CI) reflect **unstandardized mean differences between groups. IRR = Incidence rate ratio.**

**Supplemental Table 2**

*Bivariate Correlations Between Primary Variables at Baseline*

| Variable | 1. | 2. | 3. | 4. | 5. | 6. | 7. | 8. | 9. | 10. | 11. | 12. | 13. |
| --- | --- | --- | --- | --- | --- | --- | --- | --- | --- | --- | --- | --- | --- |
| 1. ADHD symptoms | — |  |  |  |  |  |  |  |  |  |  |  |  |
| 1. ODD symptoms | .69** | — |  |  |  |  |  |  |  |  |  |  |  |
| 1. BPF | .68** | .73** | — |  |  |  |  |  |  |  |  |  |  |
| 1. Depression symptoms | .56** | .47** | .72** | — |  |  |  |  |  |  |  |  |  |
| 1. Anxiety symptoms | .35** | .34** | .59** | .64** | — |  |  |  |  |  |  |  |  |
| 1. Total caregiver strain | .68** | .64** | .65** | .58** | .43** | — |  |  |  |  |  |  |  |
| 1. Objective caregiver strain | .67** | .59** | .62** | .55** | .38** | .93** | — |  |  |  |  |  |  |
| 1. Subjective internalized caregiver strain | .64** | .60** | .63** | .59** | .46** | .95** | .81** | — |  |  |  |  |  |
| 9. Subjective externalized caregiver strain | .37** | .46** | .39** | .25** | .18** | .69** | .52** | .60** | — |  |  |  |  |
| 10. Youth age | -.07** | -.03* | -.08** | -.04** | -.07** | .03* | .02 | .02 | .05** | — |  |  |  |
| 11. Parent age | -.02 | .02 | .03* | .08** | .02 | .01 | .02 | .02 | -.09** | .13** | — |  |  |
| 12. History of parent depression | .21** | .19** | .31** | .28** | .27** | .23** | .21** | .25** | .07** | -.02 | -.02 | — |  |
| 13. History of parent ADHD | .42** | .24** | .26** | .27** | .22** | .20** | .22** | .19** | .08** | .01 | -.04** | .27** | — |
| 14. Parent marital status | -.24** | -.16** | -.22** | -.19** | -.15** | -.16** | -.12** | -.16** | -.15** | -.07** | .18** | -.13** | -.23** |

*Note.* Values are based on imputed data.

**p* < .050; ***p* < .010

**Supplemental Table 3**

*Bivariate Correlations Between Primary Variables at Follow-Up*

| Variable | 1. | 2. | 3. | 4. | 5. | 6. |
| --- | --- | --- | --- | --- | --- | --- |
| 1. ADHD symptoms | — |  |  |  |  |  |
| 1. ODD symptoms | .60* | — |  |  |  |  |
| 1. BPF | .58* | .69* | — |  |  |  |
| 1. Total caregiver strain | .57* | .69* | .67* | — |  |  |
| 1. Objective caregiver strain | .53* | .64* | .64* | .92* | — |  |
| 1. Subjective internalized caregiver strain | .59* | .65* | .66* | .95* | .78* | — |
| 1. Subjective externalized caregiver strain | .32* | .55* | .41* | .78* | .56* | .70* |

*Note.* Values are based on imputed data.

**p* < .001

**Supplemental Figure 1**

***Time × ODD*** *Symptoms Interaction Predicting Objective Caregiver Strain*

*
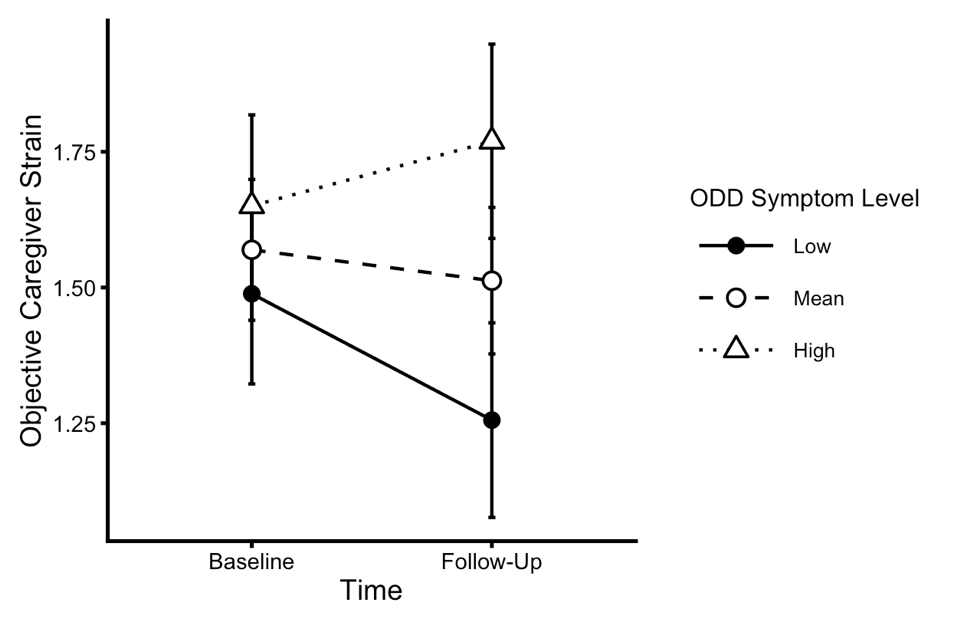
*
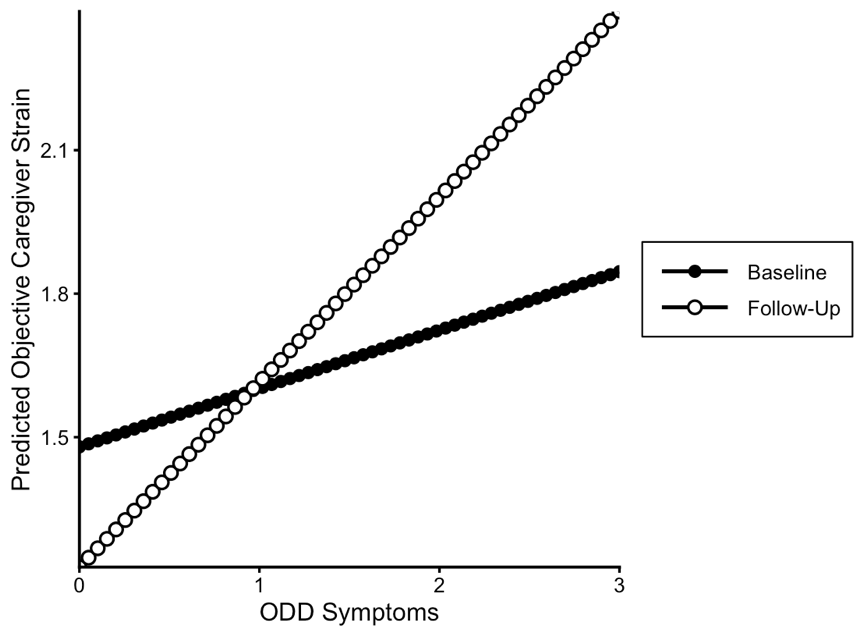


*Note.* The left panel illustrates depicts the Time × ODD symptoms interaction, showing predicted levels of objective caregiver strain across the full range of youth ODD symptoms at baseline and follow-up. The right panel displays estimated marginal means of objective caregiver strain as a function of time (baseline vs. follow-up) at low (−1 SD), mean, and high (+1 SD) levels of ODD symptoms. Error bars represent ±1 SE.

**Supplemental Figure 2**

*
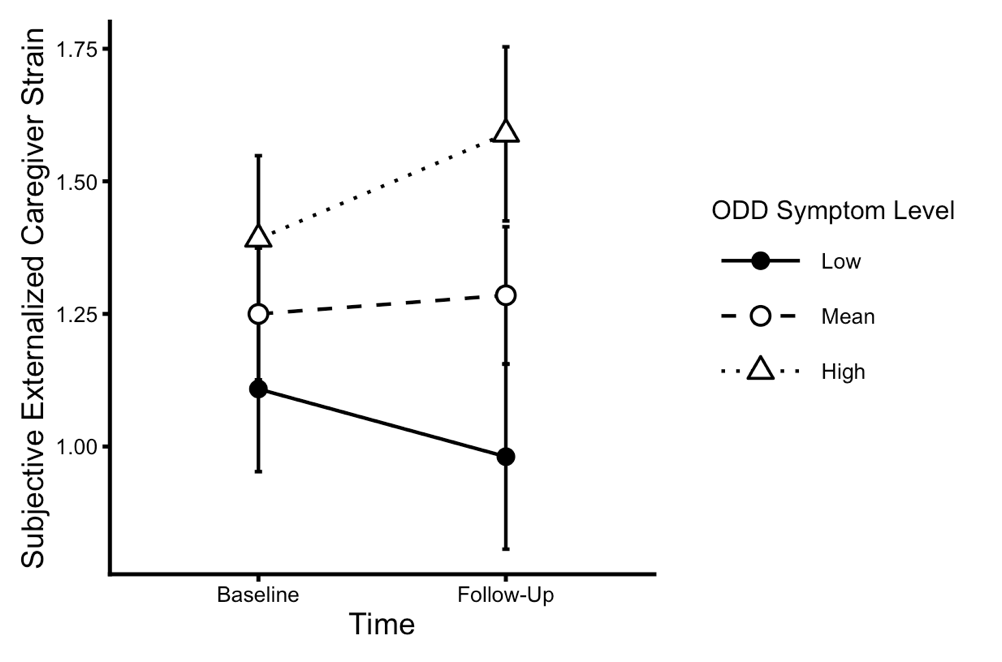

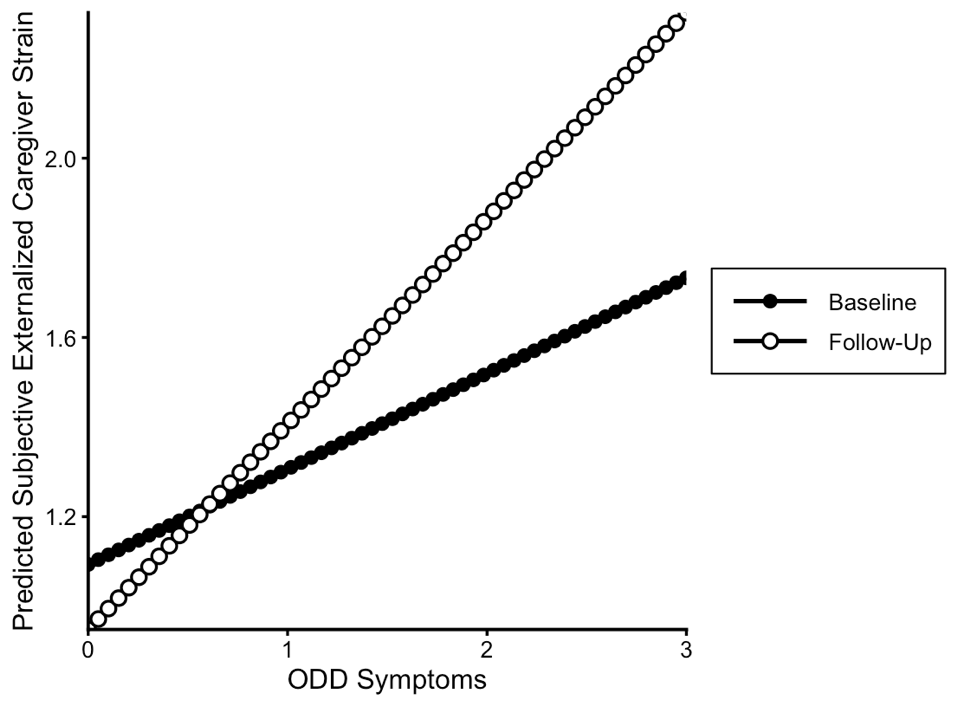
****Time × ODD Symptoms*** *Interaction Predicting Subjective Externalized Caregiver Strain*

*Note.* The left panel illustrates the Time × ODD symptoms interaction, showing predicted levels of subjective externalized caregiver strain across the full range of youth ODD symptoms at baseline and follow-up. The right panel displays estimated marginal means of subjective externalized strain as a function of time (baseline vs. follow-up) at low (−1 SD), mean, and high (+1 SD) levels of ODD symptoms. Error bars represent ±1 SE.
